# Liposomal-synthetic-cannabidiol: preliminary translational evidence of efficacy, tolerability and pharmacokinetics following repeated subcutaneous injections in two goats

**DOI:** 10.3389/fphar.2025.1689226

**Published:** 2025-11-10

**Authors:** Yael Shilo-Benjamini, Wiessam Abu Ahmad, Dinorah Barasch, Eran Lavy, Daniel Zilbersheid, Yechezkel Barenholz, Ahuva Cern

**Affiliations:** 1 Laboratory of Membrane and Liposome Research, Department of Biochemistry, Hadassah Medical School, The Hebrew University of Jerusalem, Jerusalem, Israel; 2 Koret School of Veterinary Medicine, The Robert H. Smith Faculty of Agriculture, Food and Environment, The Hebrew University of Jerusalem, Rehovot, Israel; 3 Hadassah Medical Center, The Hebrew University of Jerusalem, Jerusalem, Israel; 4 The Mass Spectrometry Unit, School of Pharmacy, The Hebrew University of Jerusalem, Jerusalem, Israel

**Keywords:** analgesia, cannabidiol, CBD, goats, liposomes, pain, pharmacokinetics, prolonged release

## Abstract

Cannabidiol (CBD), the primary non-psychoactive component of *Cannabis sativa*, has been gaining popularity as an analgesic in treatment of chronic painful conditions. Due to first-pass hepatic metabolism, oral CBD is considered to have low bioavailability. Our previous studies on dogs indicate that synthetic CBD encapsulation in liposomes facilitates controlled drug release and provides long-term CBD plasma concentrations. In the present study, liposomal CBD (5 mg/kg) was repeatedly injected subcutaneously in two goats, due to suspected pain and deterioration in quality of life (QoL). Blood samples were collected for assessment of plasma concentrations, complete blood count (CBC), and biochemical analysis before and up to 6 weeks after each injection. Efficacy was assessed by the caregivers via QoL weekly scoring, and adverse effects were monitored. A total of 14 injections were administered. No adverse effects were recorded, nor were significant changes observed in CBC and biochemistry. The CBD peak plasma concentration (C_max_) was 4.4–28.2 ng/mL, while its primary metabolite, 7-carboxy-CBD (7-COOH-CBD), was much higher (129–1,524 ng/mL), similar to those in reports of humans. The time to C_max_ and half-life of CBD were 0.25–21 and 5.1–24.2 days, respectively, and those in 7-COOH-CBD were 3–28 and 5.6–24.5 days, respectively. The concentration–time curves flattened with repeated injections. QoL improvement was observed for 4 weeks following injections. The results of this study offer clinically translatable information. Liposomal CBD injections every 6 weeks are practical, have no adverse effects, and provide long-term CBD and 7-COOH-CBD concentrations that approach steady-state concentrations over time. Additionally, liposomal CBD demonstrated remarkable efficacy in pain control and wellbeing improvement for several weeks and can potentially provide similar results in humans.

## Graphical Abstract

**Figure:**
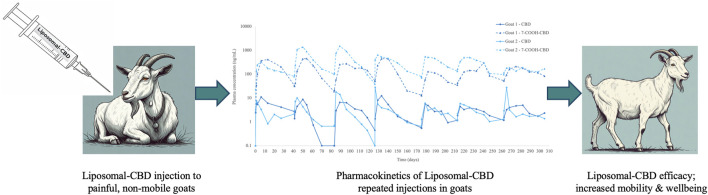
Liposomal CBD was injected to painful, non-mobile goats every 6 weeks. Plasma concentrations of CBD and its major metabolite, 7-carboxy-CBD (7-COOH-CBD), were detected for weeks, and goats showed increased mobility and improved wellbeing.

## 1 Introduction

Cannabidiol (CBD), the non-psychoactive component of *Cannabis sativa*, has gained great scientific and medical interest because of its various potential therapeutic applications (Millar et al., 2019). Additionally, CBD is considered to be of greater clinical interest due to the addictive, hallucinogenic, and toxic adverse effects of the psychoactive component tetrahydrocannabinol (THC) (Bukowska, 2024). Until now, the oral solution Epidiolex^®^ is the only US Food and Drug Administration (FDA)-approved purified CBD, indicated for refractory epilepsy (Britch et al., 2021; Silmore et al., 2021). Other suggested therapeutic applications of CBD include anti-nociceptive and anti-inflammatory characteristics, contributing to analgesia under chronic conditions in humans (Barrie and Manolios, 2017) and other species (Verrico et al., 2020; Interlandi et al., 2024). CBD is generally well tolerated even at high doses (Taylor et al., 2018; Vaughn et al., 2020). However, from a pharmaceutical perspective, CBD presents a challenge: low aqueous solubility and poor oral bioavailability (6%–13%), due to its significant first-pass liver metabolism (Perucca and Bialer, 2020). Moreover, the bioavailability is highly dependent on the fasted or fed conditions (Taylor et al., 2018; Silmore et al., 2021). The chronic nature of CBD therapeutic applications requires convenient administration routes that will overcome the drawbacks of oral administration.

Liposomal–CBD takes advantage of the highly lipophilic nature of CBD, allowing its loading into the lipid carrier. In previous research on dogs, synthetic CBD was encapsulated in large multilamellar vesicles (MLVs) to form liposomal–CBD drug product, providing an alternative subcutaneous (SC) injectable depot. This approach bypasses the liver and increases the bioavailability toward 100%, with no dependence on fasted/fed conditions (Shilo-Benjamini et al., 2022; Shilo-Benjamini et al., 2023). The liposomal–CBD formulation facilitated controlled drug release and showed long systemic exposure for more than 4 weeks, which may allow convenient, single administration per month. Additionally, a single SC injection of this formulation produced significant positive outcomes on osteoarthritic pain in dogs for several weeks (Shilo-Benjamini et al., 2023).

The preclinical investigation described here aimed to report long-term repeated administration of liposomal–CBD in a large-animal model with naturally occurring disease. Specific goals included evaluating the pharmacokinetics, metabolism, efficacy, and safety of repeated SC injections of liposomal–CBD in goats, while focusing on their translational therapeutic value.

## 2 Materials and methods

### 2.1 Animals

Two goats with naturally occurring congenital malformations (back and limbs) resulting in suspected pain were referred for an alternative analgesic treatment. The goats were housed in a rescue farm, and the caregivers sought extra-label analgesia because non-steroidal anti-inflammatory drugs (NSAIDs) did not provide sufficient pain relief. This clinical investigation was carried out as a compassion therapy in accordance with “Good Clinical Practice.” A signed informed consent was obtained from the legal guardian of the goats for participation in this investigation.

#### 2.1.1 Goat 1

A 3.5-year-old, male neutered goat, weighing 55 kg, had scoliosis and hind limb paralysis since 2 months of age. The goat was allowed to be mobile using a wheel-cart, on which he was placed for 3 h twice daily. As he grew up and increased in size and weight, the scoliosis worsened, and the goat became less active during the wheel-cart-allocated time, in which the goat leaned against the wall of his yard (Supplementary Figure S1A).

Daily NSAID injections (Finadyne, MSD Animal Health, Israel) resulted in only minor improvement in mobility and were discontinued after 8 days (3 weeks before intervention) due to possible adverse effects when used for treatment of chronic conditions.

#### 2.1.2 Goat 2

A 1-year-old, male neutered goat, weighing 31 kg, had severe kyphosis, accompanied by limb stiffness and hyperextension. Physiotherapy treatments were performed twice daily by the caregivers, which included limb exercises, followed by mobilizing the goat with support from a harness. Bruxism (teeth grinding) was the primary sign of discomfort and pain, observed during physiotherapy and walks. A secondary sign was resistance to treatments.

Daily NSAID injections (Recocam, Bimeda Animal Health Limited, Ireland; and then Rifen, Richter Pharma AG, Austria) were initiated but were discontinued after 5 days (4 weeks before intervention) when no improvement was noted.

### 2.2 Liposomal–CBD intervention

#### 2.2.1 Liposomal–CBD description

Liposomal–CBD formulation (Liposome Platform Technology; LPT-CBD) was obtained from Innocan Pharma™ (Israel). According to the product certificate of analysis, liposomal–CBD was prepared under strict aseptic conditions. Additionally, samples were submitted to Hy-Labs (Israel), a certified and accredited laboratory by the FDA, which confirmed sterility and approved the endotoxin limits (according to extravascular requirements in humans). The liposomal–CBD formulation was composed of synthetic CBD (Purisys LLC., GA, United States) with purity exceeding 98% and no detectable THC. Synthetic CBD was loaded at a concentration of 50 mg/mL into hydrogenated soy phosphatidyl choline (HSPC) liposomes (Lipoid GmbH, Germany). HSPC was used due to its good chemical stability, high phase transition temperature (53 °C), availability in large quantity as a “Good Manufacturing Practice” excipient, and prior FDA approval for other drug products, such as Doxil^®^ (Barenholz, 2012). The formulation characteristics are described in Table 1.

**TABLE 1:** Description of liposomal-synthetic-cannabidiol (L-sCBD) formulation, injected repeatedly in two goats with naturally occurring pain.

Test	Result
Appearance	“Milky” liquid in a glass vial
Appearance (light microscope)	Round particles
Total CBD assay (mg/mL)	50.0 ± 2.0
pH	7.0–7.6
Osmolality (mOsm/kg)	280–340
Sterility	No growth
Endotoxin/Pyrogen test	<5 EU/mL

#### 2.2.2 Liposomal–CBD injections

Liposomal–CBD at a dose of 5 mg/kg (0.1 mL/kg) was injected SC using a 21-gage, 1-inch needle. Injections were performed at the dorsal thoraco-lumbar area on both sides of the spine (each injection at a different site). Hair was clipped, and aseptic skin preparations using chlorhexidine and ethyl alcohol 70% were used prior to all injections.

### 2.3 Monitoring

#### 2.3.1 Pharmacokinetics

CBD and its metabolites were quantified using UHPLC-tandem mass spectrometry (LC-MS/MS), with the limits of detection (LOD) and quantification (LOQ) of 0.1 and 0.3 ng/mL, respectively. The concentration–calibration curve was prepared using naïve goat plasma. The method and materials used for quantification are reported in the Supplementary Data and Supplementary Tables S1, S2.

##### 2.3.1.1 Blood collection

Blood (1 mL) was collected from the jugular vein into 1-mL ethylenediaminetetraacetic acid (EDTA) tubes for pharmacokinetic analysis at baseline, then at 6 h, 1–2 days, 3–4 days, and subsequently weekly or every other week up to 6–7 weeks following each injection. Blood samples were centrifuged within 5 min of collection (ScanFuge Mini, ScanSpeed; LaboGene, Lillerød, Denmark; 6,200 × *g*, 10 min, at room temperature), and then plasma was frozen at −20 °C before being transferred to −80 °C until analysis.

##### 2.3.1.2 Pharmacokinetic analysis

Pharmacokinetic parameters were calculated for 6–7 weeks following each injection (depending on the timing of the last sample before the next injection) using a noncompartmental analysis with Phoenix WinNonlin (Version 8.3.5, Certara, NJ, United States). The area under the concentration–time curve (AUC) was calculated using the logarithmic trapezoidal method from the time of dosing to the last time point of plasma sampling. The mean residence time (MRT) was calculated using AUMC/AUC, where AUMC is the area under the moment curve from the time of dosing to the last measurable point. The terminal slope (λ) was estimated by linear regression through the last time points and used to calculate the terminal half-life from the following equation: half-life = 0.693/λ. Half-life values showing a good correlation were reported (R-square >0.84).

#### 2.3.2 Blood work

Blood samples were collected in EDTA tubes (1.5 mL) for complete blood count (CBC; ProCyte Dx™ Veterinary Hematology Analyzer, IDEXX Laboratories, Inc., Westbrook, Maine, United States) and in tubes containing a separator gel (CAT Serum Sep Clot Activator, Vacuette^®^, Greiner Bio-One, Kremsmünster, Austria; 2–2.5 mL) for biochemical analysis (Catalyst Dx™ Veterinary Blood Chemistry Analyzer, IDEXX Laboratories, Inc.). Samples for CBC and biochemical analysis were collected at baseline, at 3–8 days, and then at 6–7 weeks after each injection.

#### 2.3.3 Efficacy

A visual analog scale for the quality of life (QoL) score was used for assessment of goats. Out of a scale of 0–10; 0 = excellent QoL (i.e., normal goat) and 10 = poor QoL (i.e., extremely painful, non-functioning goat). A single caregiver collected data from all other caregivers and scored the QoL. Assessments were completed at baseline before the first injection and then once weekly throughout the monitoring period. The QoL assessment included goat’s appetite, vitality, mobility, playfulness, mood, presence/absence of bruxism, and cooperation/resistance during physiotherapy sessions. Validated QoL or chronic pain scales for goats were not reported; therefore, a simple analog scale, straightforward for use by caregivers, was chosen.

#### 2.3.4 Vital signs, adverse effects, and follow-up

Body weight (BW) was recorded at baseline before each injection and then at 3 weeks after injections. Physiological parameters were monitored throughout the study period: heart rate (HR) using a stethoscope, respiratory frequency (*f*_R_) by observing flank movements, and rectal temperature (RT) with a digital thermometer. The physiological parameters were measured at baseline, then at 6 h, 1–2 days, 3–4-days, and weekly until the next injection. During these examinations, goats were monitored closely for adverse effects, including local response at the injection site.

### 2.4 Statistical analysis

All parameters obtained before each injection from both goats were organized as baseline values and then compared with the parameters obtained during the weeks following each intervention (*n* = 14 for each time point). Statistical analysis was performed in RStudio, version 2023.03.1, using the lme4 and emmeans packages. To analyze the effects of different injections and time points on physiological parameters, quality-of-life scores, and blood work, linear mixed-effects models were used. These models account for both fixed effects (injection and time) and random effects (variability between individual goats). The random intercept model was used to allow for individual differences between goats, where goat ID was treated as a random effect.

To further assess the effects of time and injection, estimated marginal means were obtained using the emmeans package. Pairwise comparisons were conducted to evaluate differences across time points and between injections. Successive time-point comparisons were performed to investigate changes over time using custom contrast vectors. A *p*-value <0.05 was considered significant. Because the sample size was small, descriptive statistics are expressed as median (range; minimum–maximum).

## 3 Results

### 3.1 Pharmacokinetic data

**FIGURE 1:**
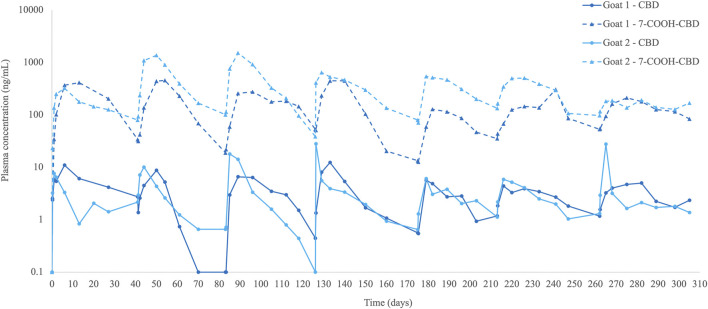
Plasma cannabidiol (CBD) concentrations (ng/mL) and its metabolite 7-carboxy-CBD (7-COOH-CBD) in two goats with naturally occurring pain before and until 6–7 weeks after multiple subcutaneous liposomal–CBD injections at 5 mg/kg (*n* = 7 injections per goat).

All calculated parameters of each injection (*n* = 14) are presented in Supplementary Table S3. CBD plasma concentrations peaked at a median of 3.5 (0.25–21) days and were 8.2 (4.4–28.2) ng/mL and above the limit of quantification until the next injection (6–7 weeks) in 6 out of 7 injections per goat (Table 2; Figure 1). After the fourth injection, trough concentrations of CBD increased, and the concentration–time curve flattened (Figure 1).

**TABLE 2:** Pharmacokinetic parameters of plasma cannabidiol (CBD) and its metabolite, 7-carboxy-CBD (7-COOH-CBD), from two goats after repeated subcutaneous injections of 5-mg/kg liposomal–CBD (each goat was administered seven injections). Blood samples were collected before and until 6–7 weeks following each injection. Data are presented as median (minimum–maximum).

Parameter	CBD		7-COOH-CBD	
	Goat 1	Goat 2	Goat 1	Goat 2
C_max_ (ng/mL)	6.6 (4.4–12.4)	10.1 (5.9–28.2)	306 (129–456)	542 (191–1,524)
T_max_ (days)	7 (3–21)	3 (0.25–4)	14 (7–28)	7 (3–21)
Half-life (days)^#^	10.3 (5.1–24.2) (*n* = 4)	14.6 (7.6–15.9) (*n* = 5)	7.8 (5.6–20.3) (*n* = 5)	13.2 (5.8–24.5) (*n* = 5)
AUC (ng·day/mL)	140 (99–230)	124 (98–189)	7,439 (2,893–9,976)	13,575 (6,557–22,813)
MRT (days)	16.2 (9.0–18.5)	11.9 (7.9–16.6)	16.8 (12.9–21.9)	16.3 (12.2–20.4)
AUC ratio of 7-COOH-CBD: CBD	NA	NA	48 (29–81)	110 (41–218)

C_max_, peak plasma concentration; T_max_, time to peak plasma concentration; AUC, area under the concentration–time curve; MRT, mean residence time; NA, not applicable.

^#^ Half-life values were included only if R square was >0.84; therefore, *n* = the number of available half-lives.

The primary metabolite detected was 7-carboxy-CBD (7-COOH-CBD), which quickly increased and was already detected 6 h after injection (first injection). 7-COOH-CBD levels peaked at a median of 14 (3–28) days, reached a concentration of 431 (129–1,524) ng/mL, and then decreased gradually, although plasma levels were still detected at 6–7 weeks after all injections (Table 2; Figure 1). The AUC ratio of 7-COOH-CBD to CBD was generally high, with a median ratio of 61 (29–218). Other metabolites, such as 6-hydroxy-CBD (6-OH-CBD) or 7-hydroxy-CBD (7-OH-CBD), were not detected in goats’ plasma.

### 3.2 Blood work

There were no significant changes from baseline in CBC or biochemistry variables throughout the monitoring period (Supplementary Table S4). These minimal changes had no clinical importance.

### 3.3 Efficacy

**FIGURE 2:**
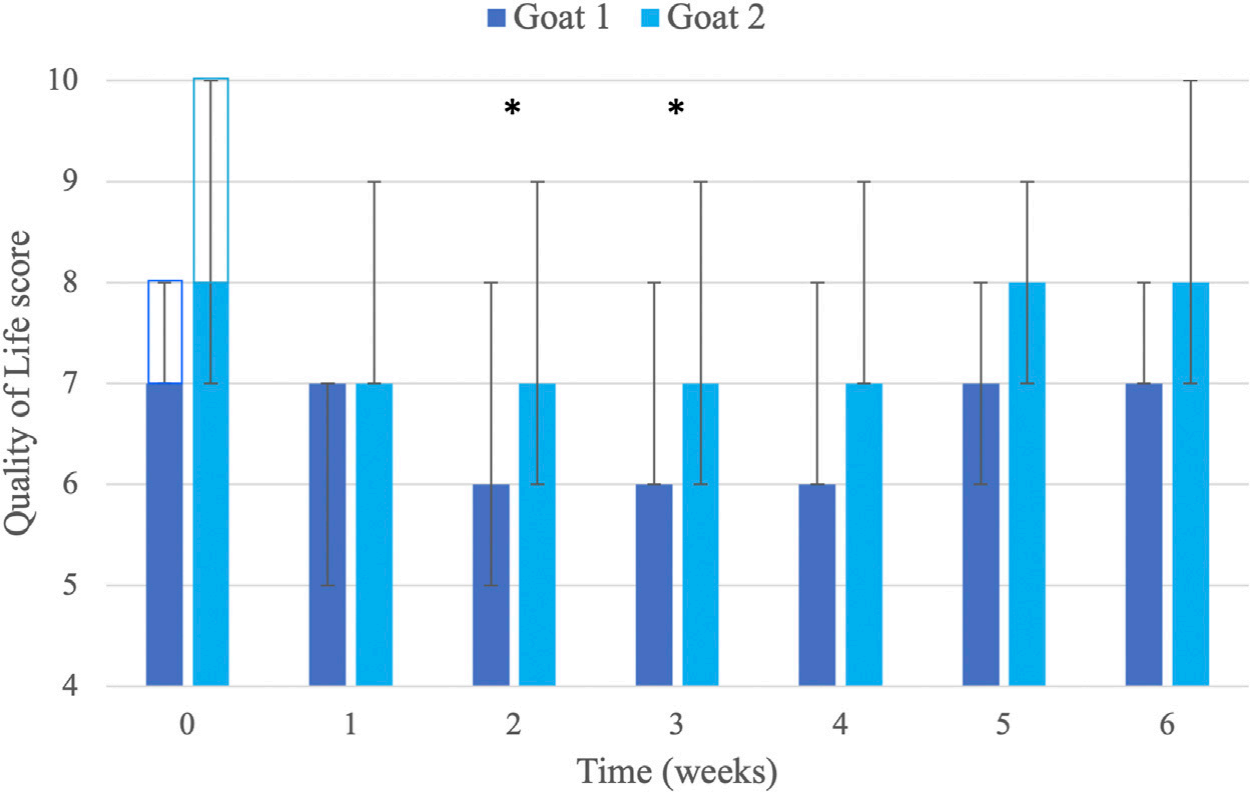
Visual analog scoring of Quality of Life (QoL; scale 0–10; 0 = excellent, no pain, normal goat; 10 = poor, extremely painful, non-functioning goat). Scores were determined by the caregivers of two goats (*n* = 7 per goat for each week) before and then weekly until 6 weeks after seven liposomal–cannabidiol (CBD) subcutaneous injections at 5 mg/kg. Data are presented as median and minimum–maximum (error bars). The clear rectangles (at time 0) represent the baseline QoL score for each goat. * Significantly improved from baseline value (*p* < 0.05; analysis performed on all data from both goats; *n* = 14 for each week).

The initial baseline QoL scores were 7 in Goat 1 and 10 in Goat 2, and the delta (difference) of scores from baseline showed consistent improvement for 1–4 weeks after each injection. Additionally, from the second injection, the initial baseline in Goat 2 showed improvement (from 10 to 8), which was maintained for the duration of the study. QoL scores were significantly decreased (i.e., QoL was improved) compared with baseline scores (8 [7–10]) on weeks 2 (6.5 [5–9]; p = 0.003) and 3 (6.5 [6–9]; p = 0.013) following injections. At weeks 1 (7 [5–9]) and 4 (7 [6–9]), there was an improvement of QoL scores, but it did not reach significance (p = 0.088; Figure 2).

#### 3.3.1 Goat 1

After the first injection, significant behavioral improvement was observed from day 4 and lasted for approximately 4 weeks and then gradually decreased in frequency until the next injection. The goat became more active and started playing with his companion goats and sheep (Supplementary Figure S1B). This playful behavior used to be common for this goat, but was not observed for at least 6 months prior to injection. Furthermore, the improvement in this activity and playfulness repeatedly resumed within a few days after subsequent injections, lasting 4–5 weeks. An exception to this improvement occurred following injection 5, due to a pressure-wound at the wheel-cart contact area. Following wound treatment and complete healing, better padding of the wheel-cart was done, which prevented this problem from reoccurring.

#### 3.3.2 Goat 2

The primary behavioral change observed by the caregivers was that 2 days after the first injection, the goat stopped grinding its teeth during physiotherapy sessions. This observation lasted for approximately 2 weeks. After the following injections, teeth grinding stopped within 1–2 days and lasted for 3–4 weeks. Additionally, the goat became more tolerant and cooperative to physiotherapy treatments and was willing to walk more during the harness walking. An improvement peak was observed a week after the fifth and seventh injections, when Goat 2 started performing small front limb jumps during harness walking. This “jumping” behavior lasted approximately 3 weeks following injections.

### 3.4 Vital signs, adverse effects, and follow up

Goats’ BW did not change significantly over time: in Goat 1, the baseline BW was 55 (53.5–56) kg and remained 55 (54.5–56) kg at 3 weeks; in Goat 2, the baseline BW was 33 (30.5–34) kg and was 32.5 (32–34) kg at 3 weeks. Physiological parameters did not undergo significant changes following injections (p > 0.05; Supplementary Table S5). Furthermore, no adverse effects were observed following any of the injections. Therefore, after the seventh liposomal–CBD injection, veterinary monitoring and CBD pharmacokinetics were discontinued. However, at the guardian’s request, both goats were continuously provided liposomal–CBD injections every 8–9 weeks (depending on their condition as assessed by the caregivers and veterinarian availability). At the time of manuscript submission, each goat was administered 17 liposomal–CBD injections over a period of 2.5 years. According to the caregivers, these injections resulted in consistent behavioral improvement, as described above, without adverse effects or blood work changes (collected every 4 months).

## 4 Discussion

### 4.1 Pharmacokinetics

Results from the present investigation suggest that repeated SC liposomal–CBD injections provide CBD and 7-COOH-CBD plasma concentrations for several weeks in a large-animal clinical model. Injectable liposomal–CBD formulation improves bioavailability and provides prolonged CBD plasma concentrations (Shilo-Benjamini et al., 2022; Shilo-Benjamini et al., 2023), as was observed in the goats. The plasma CBD profile over time was characterized by relatively constant values over the 10-month testing period.

Some C_max_ variability was observed, which is most likely attributed to variable sampling time points post-injections. Compared with six dogs administered a single dose of 5 mg/kg liposomal CBD, median C_max_ (45.2 [17.8–72.5] ng/mL) and AUC (490 [189–803] ng·day/mL) in the dogs were higher than those in the goats, while T_max_ (4 [2–14]) days] in the dogs was similar to that in the goats (Shilo-Benjamini et al., 2023). The reason for the differences in C_max_ and exposure may be related to absorption (could potentially be affected by differences in subcutaneous adipose tissue) or drug metabolism differences between the species. To the best of our knowledge, no other pharmacokinetic studies on any CBD formulation in goats are available for comparison with the liposomal–CBD depot. The results obtained are comparable to those observed with injectable sustained-release formulations administered intramuscularly, such as antipsychotics (Correll et al., 2021) or vaccines (Rahnfeld and Luciani, 2020; Jiang et al., 2023), which remain effective for weeks, suggesting slow absorption from the injection site and prolonged exposure of at least 1 month.

Following administration of Epidiolex^®^ in adults and children, the pharmacokinetics obtained were typical to that of oral administration with T_max_, most commonly observed in 3–5 h after ingestion (Britch et al., 2021). In addition, in adult healthy volunteers, C_max_ ranged from 292 to 782 ng/mL, depending on the administered dose (1,500–6,000 mg). Twenty-four hours after administration of Epidiolex^®^ in healthy volunteers, CBD levels decreased rapidly to approximately 10% of the C_max_ value, with a half-life of approximately 15 h and no dose effect (Taylor et al., 2018).

Generally, all factors influencing oral CBD absorption have not been thoroughly understood; however, the available data show that large interpatient variability exists (Silmore et al., 2021; Perucca and Bialer, 2020). Some variability is attributed to individual differences in metabolism (Perucca and Bialer, 2020). Another factor is the presence or absence of food in the gastrointestinal tract. In the fasted state, bioavailability was reduced, while in the fed state, it was increased, especially in the case of high fat content (Taylor et al., 2018; Silmore et al., 2021). It was also reported that consumption of oral CBD in the fed state produces less variability and more predictable pharmacokinetic data (Silmore et al., 2021). The explanation for this phenomenon is attributed to lymphatic transport; in the presence of a high-fat diet, highly lipophilic drugs become more associated with chylomicrons, resulting in increased intestinal absorption via the lymphatic system, thereby improving the oral bioavailability (Franco et al., 2020). Administration of injectable liposomal–CBD bypassing the liver is likely to result in increased bioavailability, as was demonstrated for dogs (Shilo-Benjamini et al., 2023), and is unlikely to be affected by the feeding state, which can lead to lower variability.

CBD plasma concentrations in the present investigation were likely affected by the dose administered, which was chosen based on the dose reported for oral administration in calves (Meyer et al., 2022) and the dose of liposomal–CBD showing efficacy in dogs (Shilo-Benjamini et al., 2023). A dose-dependent but not dose-proportional increase in CBD C_max_ and AUC was reported in studies on oral CBD administration in humans (Britch et al., 2021; Silmore et al., 2021; Devinsky et al., 2018) and in a recent meta-analysis (Moazen-Zadeh et al., 2024). A wide range of doses were reported for Epidiolex^®^ and other CBD formulations: from 20 to 6,000 mg of the total dose in adults or 2.5–40 mg/kg in children (Britch et al., 2021; Silmore et al., 2021; Taylor et al., 2019; Devinsky et al., 2018; Wheless et al., 2019). The effect of increasing the dose of liposomal-CBD on C_max_ is unknown and should be investigated in the future; however, the exposure may be limited by slow absorption from the injection site.

Qualitatively, CBD accumulation did not seem to occur, which is likely attributed to the 6-week interval between injections. Furthermore, repeated administration of liposomal-CBD over time resulted in a flatter CBD plasma concentration curve. This observation started after the fourth injection, and it was suggested that following repeated use a steady state is approached.

The primary metabolite, 7-COOH-CBD, in goats, is the same reported in humans, and the high AUC ratio of 7-COOH-CBD to CBD resembles the high ratio reported in humans (35-fold) (Tayo et al., 2020). Following absorption in humans, CBD is quickly metabolized to the active metabolite 7-OH-CBD in the liver. 7-OH-CBD is considered to possess pharmacological activity in neuronal brain tissue (Ujvary and Hanus, 2016; Zhang et al., 2024). 7-OH-CBD is further metabolized to 7-COOH-CBD, a major metabolite, with concentrations exceeding those of the parent compound. Another CBD metabolite in humans is 6-OH-CBD, which is observed at much lower concentrations and considered relatively minor metabolite owing to its low abundance (Batinic et al., 2023; Tayo et al., 2020; Zhang et al., 2024). The reason why other metabolites were not detected in the goats is unknown. One possible explanation is that dosing, and therefore CBD C_max_, was too low to produce sufficient amounts of these metabolites.

### 4.2 Analgesia

CBD was suggested to produce analgesia in humans (Murphy and Hayes, 2024; Walczynska-Dragon et al., 2024; Kulesza et al., 2024). The analgesic effect of CBD is complex as it does not bind directly to the cannabinoid receptors CB1 and CB2 but has been shown to provide analgesia through various other mechanisms of action (Kulesza et al., 2024). These include interaction with serotonergic receptors, specifically 5-HT_1A_, where CBD administration directly corrected 5-HT aberrant neurotransmission under neuropathic pain conditions. Another interaction is the activation of several transient receptor potential (TRP) ion channels, such as TRPV1, TRPV4, and TRPA1, all involved in nociception (Kulesza et al., 2024). Additional interactions were reported to occur with opioid receptors, many G protein-coupled receptors, and voltage-gated calcium channels in nociceptive neurons (Mlost et al., 2020). CBD was also reported to have an anti-inflammatory effect by inhibition of nitric oxide activity and production of several cytokines (Millar et al., 2019).

Pain is challenging to assess in ruminants because prey species tend to hide their pain, and no validated chronic pain scales in goats were reported (Tomacheuski et al., 2023). However, some behaviors suggestive of pain, observed in the goats reported here, such as modification of social behavior in Goat 1 and bruxism in Goat 2, were reported previously in the literature (Gleerup et al., 2015; Braun et al., 2020). Additionally, the remarkable improvement in these behaviors suggests excellent efficacy of liposomal-CBD in mitigating pain and increasing wellbeing.

Although CBD plasma concentrations were generally low in the goats, it is possible that CBD at low plasma concentration may still provide adequate analgesic activity, as suggested by some studies (Boehnke et al., 2022; De Gregorio et al., 2019). However, there are no pharmacokinetic data to support those studies. A recent study in humans with temporomandibular disorders reported that low CBD doses of 40 or 20 mg mixed in hydrogel and administered locally (bilateral intraoral on the masseter muscles) provided significant pain reduction, reduction in muscle tension, and alleviation of sleep bruxism compared with the vehicle control (Walczynska-Dragon et al., 2024).

Another explanation is that the analgesic effect of CBD is not reflected by CBD plasma concentrations but is related to its concentrations in the tissues. Due to its high lipophilicity, CBD is distributed quickly into body tissues, where it provides its analgesic activity (Koch et al., 2024). This is supported by its high extent of distribution in humans (Ohlsson et al., 1986) and other species (Koch et al., 2024; Samara et al., 1988; Turner et al., 2022) and by animal studies comparing CBD concentrations in plasma versus body tissues. A study on pharmacokinetics of oral CBD in rats reported that CBD plasma concentrations were 2–4 times lower than their respective brain concentrations (Bartkowiak-Wieczorek et al., 2023). A different study on rats in which CBD was administered via oral gavage reported that tissue concentrations were significantly higher in adipose tissue (116 ± 61 mg/kg tissue) than in the liver (1.0 ± 0.2 mg/kg tissue) and muscle (0.6 ± 0.2 mg/kg tissue), along with plasma, where C_max_ was approximately 1.4% of the CBD concentration in adipose tissue (Child and Tallon, 2022).

Although 7-COOH-CBD is considered the non-active metabolite in CNS disorders, such as epilepsy (Zhang et al., 2024), there is some evidence reporting that CBD metabolites 7-OH-CBD and 7-COOH-CBD can provide anti-inflammatory properties and analgesia in a mouse model (Mechoulam et al., 2010). These metabolites were synthesized and injected intraperitoneally in mice with induced ear swelling via arachidonic acid. The NSAID indomethacin (20 mg/kg intraperitoneally) was used as the positive control, and the metabolites’ vehicle was used as the negative control. Both metabolites at 40 mg/kg produced a significant decrease in ear thickness compared with the vehicle, which was comparable to indomethacin, with 7-OH-CBD producing a greater effect over 7-COOH-CBD (Mechoulam et al., 2010).

Several factors are likely contributing to the analgesic effect and increased wellbeing observed in the goats in the present study. The efficacy observed may be related to the constant concentrations that goats were exposed to, due to the controlled-release characteristics of the liposomes. Similarly, a single SC injection of liposomal–CBD provided a slow-release pharmacokinetic profile and was effective in dogs with osteoarthritis (Shilo-Benjamini et al., 2022; Shilo-Benjamini et al., 2023); thus, liposomal–CBD has the potential of providing similar positive effects in humans.

### 4.3 Safety

Multiple injections were well tolerated by the goats, without any adverse effects observed. In dogs administered with a single 5-mg/kg liposomal–CBD SC injection, significant decreases in hematocrit, albumin, total protein, creatinine, and gamma-glutamyltransferase (GGT) were reported, although all changes were clinically insignificant. Additionally, mild swelling was observed at the injection site, resolving spontaneously within several days (Shilo-Benjamini et al., 2023). These could be attributed to species differences, with dogs being more sensitive to plant-originated molecules.

Common adverse effects reported following oral CBD in humans include gastrointestinal disorders (diarrhea and loss of appetite) and nervous system disorders (somnolence, headaches, fatigue, and dizziness) (Crockett et al., 2020; Taylor et al., 2018). None of these were observed following liposomal–CBD injections in goats or dogs (Shilo-Benjamini et al., 2023). Another potential concerning effect of CBD in humans is liver toxicity with increased liver enzymes (>3-fold), even in healthy adults (Chen et al., 2024; Florian et al., 2025). It is encouraging that the liposomal-CBD used repeatedly in the present study did not result in increased liver enzymes.

### 4.4 Limitations

The limitations include the small number of goats, with only male neutered goats studied and no intact male or female goats investigated, and using a non-validated QoL scale to assess efficacy. Additionally, a major limitation is the non-blinded liposomal–CBD injections without the use of empty liposomes for control, which could have introduced a placebo effect and bias to the QoL scoring by the caregivers. However, the repeated improvement observed following all injections was unquestionable and different from any effect observed after the administration of other analgesic treatments.

## 5 Conclusion

The results of this study offer clinically translatable information. Liposomal–CBD administered SC resulted in prolonged CBD and its primary metabolite 7-COOH-CBD plasma concentrations for 6–7 weeks, which approached a steady state over time and provided high exposure in terms of AUC to the dose administered. The CBD and 7-COOH-CBD ratio in goats showed great similarity to that reported in humans. Repeated liposomal–CBD injections every 6–7 weeks are practical, have no adverse effects, and demonstrated remarkable efficacy in pain control and wellbeing improvement for several weeks.

The parenteral route provides higher bioavailability, and the sustained plasma profile provides relatively constant plasma levels over a period of weeks, without daily fluctuations typical to oral CBD administration. The liposomal–CBD formulation, which exhibits a prolonged CBD plasma profile, together with prolonged effects for several weeks after each administration, may suggest a convenient drug product providing non-addictive analgesia with a different mode of action, potentially used alongside conventional pain killers, such as NSAIDs. Thus, future directions of liposomal–CBD studies should include translation and investigation in human medicine.

## Data Availability

The original contributions presented in the study are included in the article/Supplementary Material; further inquiries can be directed to the corresponding author.
