# Supplementary material for: Liposomal-synthetic-cannabidiol: preliminary translational evidence of efficacy, tolerability and pharmacokinetics following repeated subcutaneous injections in two goats
Source: Front Pharmacol. 2025 Nov 10;16:1689226. doi: 10.3389/fphar.2025.1689226 (PMC12641115; doi:10.3389/fphar.2025.1689226)

Supplementary material

1 Supplementary Data

Quantification of CBD and its metabolites in plasma

The materials used included: LC/MS-grade Acetonitrile (ACN), Methanol (MeOH) and water were purchased from Biolab Ltd. (Jerusalem, Israel). LC/MS-grade Formic acid (FA) was purchased from Fisher Chemical™ Optima™ (USA).

UHPLC-MS/MS analyses were conducted on a Sciex (Framingham, MA, USA) Triple Quad™ 5500 mass spectrometer coupled with a Shimadzu UHPLC System (Kyoto, Japan). The chromatographic separations were performed on a CORTECS® (Waters Corp., Milford, MA, USA) column (C18, 2.7 µm particle size, 100 x 2.1 mm), protected by a VanGuard® (Waters Corp., Milford, MA, USA) pre-column. The injection volume was 10 µL, the oven temperature was maintained at 40°C and the autosampler tray temperature was maintained at 5°C. Gradient elution mobile phases consisted of phase A (0.1% FA in water) and phase B (acetonitrile). Gradient elution (300 µL/minute) was held at 15% B for the first 0.5 minutes, followed by a linear increase towards 60% B in 6.5 minutes, a linear increase towards 75% B in 3 minutes, a linear increase towards 98% B in 2 minutes and held at 98% B for 3 minutes, before reequilibrating for 3 minutes at initial conditions.

CBD and its metabolites were detected and quantified in positive and negative ion mode using electron spray ionization (ESI) and multiple reaction monitoring (MRM) mode of acquisition. CBG was used as internal standard (IS). Their transitions are shown in Supplementary Tables S1 and S2. The TurboIonspray® probe temperature was set at 500°C with the ion spray voltage at 5500 V (for positive mode) and -4500V (for negative mode). The curtain gas was set at 25.0 psi. The nebulizer gas (Gas 1) was set to 50 psi, the turbo heater gas (Gas 2) was set to 60 psi and the collision gas (CAD) was set to 8 psi. The entrance potential (EP) was set at 10 V and -10V, respectively. The collision energy potentials (CE), collision cell exit potentials (CXP) and declustering potentials (DP) for the monitored transitions are given in Supplementary Tables S1 and S2. The dwell time was 15 milliseconds. Data acquisition was performed using Analyst 1.6.3 software and data was analyzed using MutiQuant 2.1 software, both distributed by Sciex. For CBD and its metabolites, the limits of detection (LOD) and quantification (LOQ) were 0.1 and 0.3 ng/mL, respectively.

2 Supplementary Tables and Figures

2.1 Supplementary Tables

Supplementary Table S1. Plasma concentration analysis of cannabidiol (CBD) by UHPLC-MS/MS. Cannabigerol (CBG) was used as internal standard (IS). Multiple reaction monitoring (MRM) transitions and parameters for CBD and CBG (IS) in positive ion mode.

Name	Precursor (*m*/*z*)	Product (*m*/*z*)		DP (V)	CE (eV)	CXP (V)	Rt (min)
**CBD**	315.1	Quantifier	123.1	40	43	18	10.65
		Qualifier	193.1	40	34	20	
**CBG**	317.1	Quantifier	123.1	80	43	14	10.55
		Qualifier	193.1	80	23	24	

m/z: mass to charge ratio; DP: declustering potential; CE: collision energy; CXP: collision cell exit potential; V: volts; eV: electronvolts; Rt: retention time.

Supplementary Table S2. Plama concentration analysis of cannabidiol (CBD) metabolites by UHPLC-MS/MS. Cannabigerol (CBG) was used as internal standard (IS). Multiple reaction monitoring (MRM) transitions and parameters for CBD metabolites and CBG (IS) in negative ion mode.

Name	Precursor (*m*/*z*)	Product (*m*/*z*)		DP (V)	CE (eV)	CXP (V)	Rt (min)
**7-OH-CBD**	329.1	Quantifier	268.2	-40	-36	-11	7.65
		Qualifier	299.0	-40	-22	-29	
**6α-OH-CBD**	329.1	Quantifier	158.2	-130	-40	-21	7.48
		Qualifier	173.2	-130	-34	-21	
**6β-OH-CBD**	329.1	Quantifier	173.2	-130	-34	-21	7.78
		Qualifier	158.2	-130	-40	-21	
**7-COOH-CBD**	343.0	Quantifier	231.0	-55	-34	-23	7.50
		Qualifier	179.1	-55	-32	-17	
**CBG**	315.1	Quantifier	191.2	-80	-32	-7	10.55

m/z: mass to charge ratio; DP: declustering potential; CE: collision energy; CXP: collision cell exit potential; V: volts; eV: electronvolts; Rt: retention time.

Supplementary Table S3. Calculated pharmacokinetic parameters of plasma cannabidiol (CBD) and its metabolite, 7-carboxy-CBD (7-COOH-CBD) following repeated subcutaneous injections of 5 mg/kg liposomal-CBD (*n* = 14 injections). Plasma samples were collected from two goats before and up to 6-7 weeks following each injection (each goat was administered 7 injections). Parameters were calculated for each injection separately.

	Injection number	Goat number	C_{max} (ng/mL)	T_{max} (days)	Half-life (days)	AUC (ng·d/mL)	MRT (days)	7-COOH-CBD to CBD AUC ratio
**CBD**	**1**	1	11	7	24.2	230	16.7	43
		2	7.7	1	NC	98	16.6	71
	**2**	1	8.8	7	NC	106	9.0	81
		2	10.1	3	12.1	104	10.5	218
	**3**	1	6.6	7	5.1	152	16.2	49
		2	18.0	2	7.6	189	7.9	121
	**4**	1	12.4	7	9.0	175	11.9	50
		2	28.2	0.25	14.6	150	11.9	102
	**5**	1	5.7	4	NC	99	14.4	29
		2	6.1	4	15.9	106	15.9	118
	**6**	1	4.4	3	11.7	123	18.0	48
		2	5.9	3	15.1	124	15.1	110
	**7**	1	5.0	21	NC	140	18.5	43
		2	27.7	3	NC	158	11.5	41
**CBD Median**			**8.2**	**3.5**	**12.1**	**132**	**14.8**	**61**
**7-COOH-CBD**	**1**	1	413	14	6.0	9,976	16.7	
		2	324	7	24.5	6,962	16.4	
	**2**	1	456	14	7.8	8,554	14.3	
		2	1,374	7	9.0	22,739	12.7	

**3**	1	276	14	5.6	7,439	19.4
	2	1,524	7	5.8	22,813	12.2
**4**	1	448	14	14.0	8,668	12.9
	2	645	3	13.2	15,238	16.3
**5**	1	129	7	NC	2,893	16.8
	2	542	4	13.7	12,566	15.6
**6**	1	306	28	20.3	5,899	21.9
	2	504	14	NC	13,575	17.5
**7**	1	212	14	NC	6,078	19.8
	2	191	21	NC	6,557	20.4
**7-COOH-CBD Median**		**431**	**14**	**11.1**	**8611**	**16.6**

C_{max}, peak plasma concentration; T_{max}, time to maximum plasma concentration; AUC, area under the concentration–time curve; MRT, mean residence time; NC, not calculated (R square < 0.84).

Supplementary Table S4. Complete blood count and biochemistry panel performed in two goats with naturally-occurring pain, before (baseline; including baselines at 6-7 weeks from the former injection; *n* = 14) and after 3-8 days (*n* = 14) from repeated liposomal-cannabidiol (CBD) subcutaneous injection at 5 mg/kg. Data are presented as median (minimum-maximum).

Parameter	Reference range	Baseline	3-8 days
**Hematology**			
Red blood cells ($10^6/\mu L$)	10.32-23.43	23.35 (20.9-32.3)	22.0 (18.5-26.7)
Hematocrit (%)	22.0-39.0	29.9 (24.1-38.3)	28.2 (21.1-34.9)
Hemoglobin (g/dL)	8.9-13.8	10.2 (9.3-13.2)	10.2 (8.2-11.7)
Mean corpuscular volume (MCV; fL)	14.0-22.3	12.3 (11.1-16.3)	12.6 (10.8-15.1)
Mean corpuscular hemoglobin (MCH; pg)	5.0-7.0	4.4 (4.1-4.8)	4.4 (4.1-4.7)
Mean corpuscular hemoglobin concentration (MCHC; g/dL)	32.0-34.0	35.4 (28.2-39.8)	34.8 (30.7-40.5)

Reticulocytes (10^9/L)	0.0-15.0	1.05 (0-8.7)	2.1 (0-15)
Reticulocytes (%)	NA	0 (0)	0 (0-0.1)
White blood cells (10^3/μL)	6.03-19.58	10.46 (7.37-21.81)	10.11 (8.01-14.35)
Neutrophils (10^3/μL)	1.72-10.61	3.14 (2.29-9.4)	3.24 (1.78-6.29)
Lymphocytes (10^3/μL)	2.68-11.54	6.32 (3.65-9.19)	6.31 (5.1-9.58)
Monocytes (10^3/μL)	0.06-0.89	0.56 (0.32-1.13)	0.48 (0.25-1.59)
Eosinophils (10^3/μL)	0.03-1.29	0.25 (0.03-2.13)	0.31 (0.04-1.04)
Basophils (10^3/μL)	0.00-0.24	0.025 (0-0.16)	0.02 (0-0.12)
Neutrophils (%)	NA	30.2 (21.3-50.4)	27.6 (19.7-47.8)
Lymphocytes (%)	NA	61.65 (37.6-69.9)	63.5 (49.0-69.6)
Monocytes (%)	NA	4.8 (2.1-11.6)	5.0 (1.9-15.8)
Eosinophils (%)	NA	2.15 (0.3-9.8)	2.4 (0.4-8.7)
Basophils (%)	NA	0.25 (0-1.4)	0.2 (0-1.2)
Platelets (10^3/μL)	246-912	417 (220-566)	362 (202-588)
Mean platelet volume (MPV; fL)	NA	8.5 (7.6-9.2)	7.9 (7.5-8.8)
**Biochemistry**			
Alanine aminotransferase (ALT; IU/L)	23-44	25 (16-37)	26 (16-40)
Alkaline phosphatase (ALP; IU/L)	75-228	358 (225-614)	333 (220-626)
Gamma-glutamyl transferase (GGT; IU/L)	60-101	41 (32-61)	40 (33-50)
Amylase (U/L)	1-30	11 (5-19)	10 (5-28)
Lipase (U/L)	NA	205 (103-385)	182 (129-434)
Cholesterol (mg/dL)	63-108	69 (37-93)	70 (57-95)
Total bilirubin (mg/dL)	0.1-0.3	0.2 (0.1-0.4)	0.2 (0.1-0.4)
Glucose (mg/dL)	54-93	60 (49-70)	61 (48-69)
Albumin (g/dL)	2.8-3.8	3.2 (2.7-3.9)	3.1 (2.4-3.5)
Globulin (g/dL)	NA	4.2 (3.8-5.0)	4.2 (3.8-4.8)
Total protein (g/dL)	6.4-7.8	7.5 (6.6-8.5)	7.3 (6.4-7.8)
Urea (mg/dL)	10-21	11 (6-16)	10 (4-16)
Creatinine (mg/dL)	0.6-1.4	0.7 (0.5-0.8)	0.7 (0.5-0.8)
Phosphate (mg/dL)	4.2-7.6	5.0 (2.3-6.9)	5.1 (4.0-6.3)
Calcium (mg/dL)	8.2-9.8	9.0 (8.6-9.7)	9.0 (8.1-9.5)

Sodium (mmol/L)	NA	151 (144-154)	149 (146-155)
Potassium (mmol/L)	NA	4.6 (4.0-5.7)	4.6 (3.8-5.8)
Chloride (mmol/L)	NA	107 (103-109)	108 (102-110)
Osmolality (mmol/kg)	NA	293 (287-306)	295 (280-304)

NA, not applicable

Supplementary Table S5. Physiologic parameters recorded from two goats with naturally-occurring pain, before (baseline; including baselines at 6-7 weeks from the former injection) and up to 5 weeks from repeated subcutaneous injection of 5 mg/kg liposomal-cannabidiol (CBD). Each goat was administered 7 injections. Data is presented as median (minimum-maximum).

Time	HR (bpm)	f_R (rpm)	RT (°C)
Baseline	94 (72-108)	36 (24-68)	38.8 (37.8-39.6)
6 hours	88 (72-104)	36 (20-48)	38.7 (38.1-39.8)
1-2 days	92 (72-100)	30 (28-36)	38.7 (38.3-39.1)
3-4 days	88 (80-92)	34 (20-68)	38.5 (37.8-39.3)
1 week	86 (76-104)	36 (20-52)	38.7 (37.9-39.5)
2 weeks	88 (72-112)	36 (24-52)	38.7 (37.5-39.1)
3 weeks	86 (80-104)	40 (20-72)	38.4 (37.8-39.8)
4 weeks	86 (72-108)	42 (28-48)	38.6 (38.1-39.4)
5 weeks	86 (80-96)	40 (28-60)	38.1 (38.0-38.9)

HR, heart rate; f_R, respiratory frequency; RT, rectal temperature*; bpm, beats per minute; rpm, respirations per minute.
* Normal RT in goats is 38.5-39.5°C.

2.2 Supplementary Figures

Supplementary Figure S1. Activity of Goat 1. (**A**) before liposomal-cannabidiol (CBD) injection; not active, leaning on yard wall, and (**B**) several days after 5 mg/kg liposomal-CBD subcutaneous injection; active and playful.

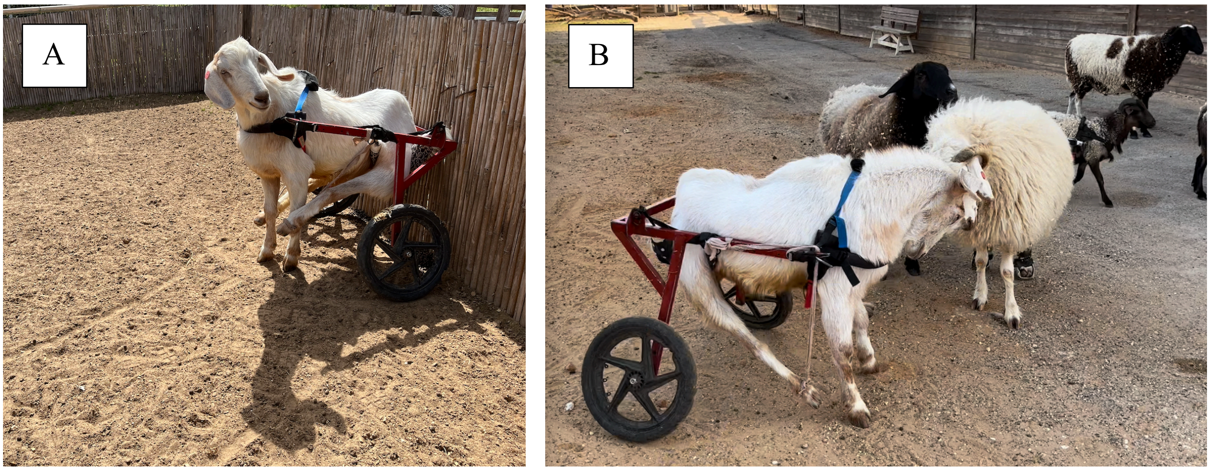

Supplement: Supplementary file 1 [file DataSheet1.pdf]
